# Reassessing *Schistosoma* worms: the overlooked role in host pathology and disease elimination

**DOI:** 10.1186/s40249-025-01298-5

**Published:** 2025-03-26

**Authors:** Haoran Zhong, Zhiqiang Fu, Jinming Liu, Yamei Jin

**Affiliations:** National Reference Laboratory for Animal Schistosomiasis, Key Laboratory of Animal Parasitology of Ministry of Agriculture and Rural Affairs, Shanghai Veterinary Research Institute, Chinese Academy of Agricultural Sciences, Shanghai, People’s Republic of China

**Keywords:** Schistosomiasis, Single-sex infection, Host-pathogen interaction, Diagnostic and therapeutic strategy

## Abstract

**Background:**

Schistosomiasis, a neglected tropical disease, remains a pressing global health challenge, hindering progress toward achievement of the Sustainable Development Goals (SDGs) in endemic regions. Despite advances in control strategies, including preventive chemotherapy and integrated measures, the elimination of schistosomiasis remains an elusive goal. Current understanding of schistosomiasis pathogenesis has largely focused on egg-induced pathology, while the contributions of schistosome worms to disease progression are relatively underexplored. The objective of this article is to highlight the critical, yet overlooked, role of schistosome worms in disease progression and to advocate for a broader research focus on their direct impact on host pathology and efforts towards disease elimination.

**Main text:**

Single-sex schistosome infections, which may occur in low-transmission areas, deserve greater attention as they evade traditional egg-based diagnostics. These infections also provide a valuable model to explore the direct contributions of worms to host pathology. Recent studies suggest that schistosome worms, via their excretory-secretory products (ESPs), contribute to liver inflammation, fibrosis, and immune modulation independent of egg deposition. Understanding the interactions between worms and hosts is essential for elucidating their role in disease progression. Furthermore, the potential similarities between schistosome ESPs and those of carcinogenic trematodes highlight the need for further investigation into their long-term impact on host health and schistosomiasis pathology.

**Conclusions:**

Expanding the focus of schistosomiasis research to include the role of schistosome worms is essential for advancing diagnostic and therapeutic strategies. By incorporating single-sex infection models and targeting worm-derived molecules, it is possible to uncover the overlooked aspects of schistosomiasis pathogenesis, improve diagnostic accuracy, and support global elimination efforts, thereby contributing to the realization of the SDGs.

**Graphical Abstract:**

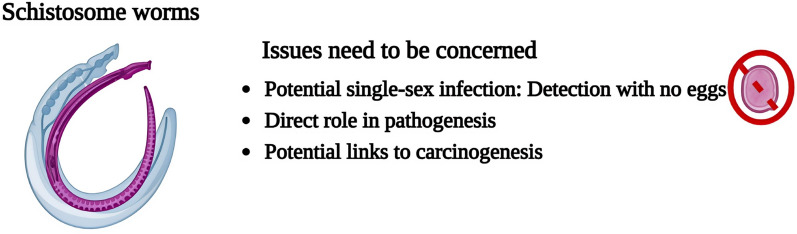

## Background

Schistosomiasis, a neglected tropical disease (NTD), remains a significant public health challenge, more than 800 million people worldwide live in areas endemic for schistosomiasis, particularly in low- and middle-income countries [1]. In certain regions, such as China, decades of integrated control measures have successfully reduced the prevalence of the disease to historically low levels [2]. Under the strategic initiatives and technical guidance provided by the World Health Organization (WHO), significant progress has been made in controlling disease transmission in various endemic regions, particularly through the implementation of large-scale preventive chemotherapy (PC) programs [3]. However, despite these achievements, the goal of eliminating schistosomiasis as a public health problem, as outlined in the WHO 2021–2030 Roadmap for NTDs, remains a challenging objective [4].

Schistosome eggs are the primary agents of schistosomiasis-related pathology and disease transmission [5]. In field diagnostics, the detection of eggs in feces or urine remains one of the most practical and reliable methods for confirming infection. The introduction of praziquantel (PZQ), a highly effective worm-killing drug, further shifted the research focus toward egg-induced pathology, resulting in the diminished attention to the role of schistosome worms. However, exploring the influence of worms is essential for addressing knowledge gaps, enhancing our understanding of disease mechanisms, and refining control strategies. This commentary aims to shed light on the overlooked contributions of schistosome worms and provide a new perspective on accelerating schistosomiasis elimination through a deeper understanding of host-parasite interactions.

### Diagnostic and therapeutic challenges of single-sex schistosome infections

Schistosomes are dioecious parasites with a complex life cycle that alternates between mammalian definitive hosts and snail intermediate hosts, involving both asexual and sexual reproduction through stages such as eggs, miracidia, mother sporocysts, daughter sporocysts, cercariae, schistosomula and adult worms [6]. In natural environments, typically only one single miracidium can successfully develop within a snail, leading to the release of single-sex cercariae [7]. Field investigations, including sentinel mouse experiments and surveys of wild snail populations, have revealed a notable prevalence of single-sex infections in both snails and mammalian hosts [8]. Due to the decline in wild snail populations and the reduced prevalence of schistosome infections resulting from successful PC programs, single-sex infections may become more common. These infections present new challenges to the diagnosis and treatment of schistosomiasis, potentially undermining progress toward the elimination of schistosomiasis. Unlike infections with paired schistosomes, hosts with single-sex infections do not produce mature eggs, which limits the effectiveness of traditional egg-based diagnostic methods. Negative stool tests in individuals or animals, alongside positive results for antibodies, nucleic acids, or point-of-care circulating cathodic antigen (POC-CCA), are frequently misinterpreted as indications of prior exposure, cross-reactivity, false-positive reactions, or the degradation products of worms, which increases the risk of undiagnosed cases [9–12]. Moreover, single-sex schistosomes can persist in the host for at least one year, and if the host later encounters opposite-sex cercariae, the worms can still mate and produce viable eggs [13]. Laboratory studies further indicate that single-sex worms exhibit reduced sensitivity to the current standard PZQ dosage (400 mg/kg), with a significant proportion surviving the treatment, although the mechanisms underlying this reduced sensitivity remain unclear [14]. These diagnostic and therapeutic challenges associated with single-sex infections highlight the urgent need for improved tools and strategies to safeguard progress toward the elimination of schistosomiasis.

### Direct role of schistosome worms in pathogenesis

Schistosome eggs are widely recognized as the primary drivers of schistosomiasis-related pathology. In *Schistosoma japonicum* and *S. mansoni* infections, eggs become lodged in the host liver, triggering intense immune responses and the formation of hepatic granulomas. These granulomas form as a protective response to egg antigens, primarily mediated by Th2-dominant immune responses and the activation of hepatic stellate cells, which play a pivotal role in fibrosis by promoting collagen deposition. Over time, unresolved granulomas transition into fibrotic plaques, resulting in chronic hepatic fibrosis, which is a leading cause of morbidity and mortality in schistosomiasis patients [15]. Notably, recent studies have revealed that, beyond the well-established pathogenic role of eggs, schistosome worms can directly regulate hepatic fibrosis through the secretion of bioactive molecules, such as extracellular vesicles (EVs) [16]. These findings reveal a distinct mechanism of worm-host interaction and provide a novel perspective on the role of worms in modulating host pathology.

To investigate the direct effects of schistosome worms on host pathology, single-sex infection models provide critical insights. As highlighted in recent studies, single-sex schistosomes can persist in hosts for extended periods, without causing overt clinical symptoms detectable by conventional diagnostic methods [13]. However, mouse models infected with *S. japonicum* or *S. mansoni* for 8–9 months have demonstrated significant liver pathology even in the absence of egg deposition. These pathologies include liver fibrosis, characterized by inflammatory cell infiltration, collagen fiber proliferation and pigment deposition. Notably, single-sex male infections induce more pronounced effects than single-sex female infections [17, 18]. One possible explanation for this phenomenon is that male-only infections lead to a stronger immune activation than female-only infections, including upregulation of genes associated with cell cycle processes and DNA organization. This heightened immune response is linked to increased liver damage, including fibrosis, suggesting that male schistosomes, due to their stronger immunogenicity, may provoke a more intense inflammatory response [19, 20]. Furthermore, single-sex infections have been shown to modulate host immune responses by inducing both pro-inflammatory and regulatory immune pathways in the liver, creating a dynamic balance between inflammation and immune regulation. This dual modulation reflects the complex interactions between single-sex schistosomes and the host immune system, potentially influencing the progression of hepatic fibrosis [17].

Given that single-sex schistosomes (particularly single-sex males) do not produce eggs, the observed liver pathology must be directly linked to worm-derived molecules. Adult worms, even in single-sex infections, secrete and excrete a range of bioactive molecules, including proteins, lipids, and nucleic acids, which can be released either as excretory-secretory products (ESPs) or within EVs. These molecules, along with worm degradation products, are transported via the host bloodstream to the liver, where they likely contribute to the establishment of a pro-inflammatory microenvironment, driving pathological changes. Furthermore, when single-sex-infected hosts are subsequently exposed to opposite-sex cercariae, resulting in bisexual infections, the liver pathology becomes more severe than that observed in typical bisexual infections [17, 21]. This phenomenon highlights the potential role of worms in priming the liver for enhanced egg-induced pathology and emphasizes the need to investigate worm-derived factors in the intricate host-parasite dynamics of schistosomiasis.

Most studies on schistosomiasis-related hepatic fibrosis have primarily focused on the role of eggs. Egg-derived EVs and other bioactive molecules have been shown to both promote and inhibit fibrosis, contributing to a chronic dynamic balance in which fibrotic progression often persists over regression [22, 23]. However, it remains challenging to conclusively determine whether such substances originate exclusively from schistosome eggs or worms. Experimental approaches designed to alter the abundance of these substances in the host liver for functional analysis cannot definitively attribute the observed effects to either eggs or worms, further complicating the understanding of their respective roles. This ambiguity has often led to an underestimation of the worm's detrimental role in host pathology.

### Potential links to carcinogenesis

Currently, aside from *S. haematobium*, which has been definitively linked to bladder cancer, the direct relationship between other schistosome species, such as *S. japonicum* and *S. mansoni*, and cancer remains uncertain [24–26]. In contrast, *Clonorchis sinensis* and *Opisthorchis viverrini*, both members of the same class Trematoda, have been classified by the WHO as Group 1 carcinogens. Extensive research has demonstrated that these liver flukes, during their prolonged residence in the bile ducts, secrete ESPs that damage the integrity of biliary epithelial cells, trigger chronic inflammation, and ultimately drive the malignant transformation of biliary epithelium. Additionally, the bile duct damage resulting from their parasitism is strongly associated with repeated mechanical injury and immune dysregulation associated with chronic infections—factors that significantly contribute to the carcinogenic process [27].

Given the evolutionary conservation of certain proteins among trematode species, it is plausible that ESPs from *S. japonicum* and *S. mansoni* may contain molecules with latent carcinogenic potential. However, since the pathogenicity of these species is primarily attributed to their eggs and their role in inducing granulomas and fibrosis, definitive evidence supporting the carcinogenic effects of their ESPs—similar to those observed in liver flukes—remains absent in the current literature. Furthermore, late-stage schistosomiasis patients are typically treated with PZQ immediately upon diagnosis, with subsequent therapeutic and research efforts primarily focused on fibrosis regression [28]. This focus inadvertently overlooks the potential long-term impacts that worm-derived ESPs may have already exerted on the host, leaving a critical gap in understanding the broader implications of schistosome-host interactions.

### Future perspectives

#### Challenges in diagnosing single-sex worm infections

Given the underexplored role of schistosome worms, it is necessary to address their relevance, particularly in low-prevalence regions where single-sex worm infections may require additional consideration. These infections evade detection by egg-based parasitological methods, emphasizing the importance of identifying diagnostic targets from worm-derived ESPs (whether from paired or single-sex worms), such as the currently utilized CCA and circulating anodic antigen (CAA) [29, 30]. However, challenges such as antigen stability and cross-reactivity with other parasites need to be addressed. Ensuring antigen stability under varying conditions and minimizing cross-reactivity are key factors for reliable diagnostics. Ongoing efforts aim to optimize antigen preparation, improve specificity, and validate clinical utility to enhance diagnostic accuracy and support schistosomiasis elimination.

### Understanding worm contributions to host pathology

Beyond their diagnostic implications, these overlooked single-sex infections offer a unique opportunity to better understand the direct contributions of schistosome worms to host pathology. While most research on schistosome pathogenesis (especially *S. japonicum* and *S. mansoni*) focuses on egg-mediated effects, the contribution of worms to long-term host-parasite interactions requires more attention. Single-sex infection models offer valuable tools for exploring the direct effects of worms on the host, particularly in the absence of egg deposition. For example, Langenberg and colleagues [31] demonstrated the potential of controlled human infection models with male *S. mansoni* cercariae, highlighting how these models can accelerate drug and vaccine development while also shedding light on immune responses in the absence of egg-induced pathology. These models have been extensively studied in murine systems, and early evidence from human clinical studies involving single-sex male worm infections suggests promising potential for broader applications. By expanding research in this area, the field may uncover critical insights into disease mechanisms and identify novel therapeutic targets, ultimately paving the way for more comprehensive strategies to combat schistosomiasis.

## Conclusions

Schistosomiasis remains a pressing global health challenge, hindering progress toward the Sustainable Development Goals in endemic regions. While elimination efforts have achieved notable success, understanding the disease's pathogenesis beyond the egg-induced effects is essential. Schistosome worms, particularly through ESPs, represent a critical yet underexplored aspect of host–pathogen interactions. Evidence from single-sex infection models emphasizes that worms can independently drive pathology, and the potential for single-sex infections may increase in low-prevalence regions, further complicating disease detection. The development of precise diagnostic tools targeting worm-derived ESPs is promising, especially in areas where traditional egg-based methods fall short. Such advancements will not only enhance diagnostic accuracy but also open new avenues for identifying therapeutic targets. Emphasizing the role of schistosome worms in research and control strategies will deepen our understanding of host-parasite dynamics and contribute significantly to the global effort to fight against schistosomiasis and mitigate its long-term health impacts.

## Data Availability

Not applicable.

## Abbreviations

CAA: Circulating anodic antigen
CCA: Circulating cathodic antigen
ESPs: Excretory-secretory products
EVs: Extracellular vesicles
NTD: Neglected tropical disease
PC: Preventive chemotherapy
POC: Point-of-care
PZQ: Praziquantel
SDGs: Sustainable development goals
WHO: World Health Organization

## References

[1] Colley DG, Bustinduy AL, Secor WE, King CH. Human schistosomiasis. Lancet. 2014;383:2253–64. 24698483 10.1016/S0140-6736(13)61949-2 PMC4672382

[2] Zhang L, He J, Yang F, Dang H, Li Y, Guo S, et al. Progress of schistosomiasis control in People’s Republic of China in 2023. Zhongguo Xue Xi Chong Bing Fang Zhi Za Zhi. 2024;36:221–7. 38952305 10.16250/j.32.1374.2024116

[3] McManus DP, Dunne DW, Sacko M, Utzinger J, Vennervald BJ, Zhou XN. Schistosomiasis. Nat Re Dis Primers. 2018;4:13. 10.1038/s41572-018-0013-8 30093684

[4] Lo NC, Bezerra FSM, Colley DG, Fleming FM, Homeida M, Kabatereine N, et al. Review of 2022 WHO guidelines on the control and elimination of schistosomiasis. Lancet Infect Dis. 2022;22:e327–35. 35594896 10.1016/S1473-3099(22)00221-3

[5] Nation CS, Da’dara AA, Marchant JK, Skelly PJ. Schistosome migration in the definitive host. PLoS Negl Trop Dis. 2020;14: e0007951. 32240157 10.1371/journal.pntd.0007951 PMC7117656

[6] Wheater PR, Wilson RA. *Schistosoma mansoni*: a histological study of migration in the laboratory mouse. Parasitology. 1979;79:49–62. 542321 10.1017/s0031182000051970

[7] Beltran S, Boissier J. Male-biased sex ratio: why and what consequences for the genus *Schistosoma*? Trends Parasitol. 2010;26:63–9. 20006552 10.1016/j.pt.2009.11.003

[8] Shi HP, Lu DB, Shen L, Shi T, Gu J. Single- or mixed-sex *Schistosoma japonicum* infections of intermediate host snails in hilly areas of Anhui. China Parasitol Res. 2014;113:717–21. 24292605 10.1007/s00436-013-3700-0

[9] Lu DB, Deng Y, Ding H, Liang YS, Webster JP. Single-sex schistosome infections of definitive hosts: Implications for epidemiology and disease control in a changing world. PLoS Pathog. 2018;14: e1006817. 29494686 10.1371/journal.ppat.1006817 PMC5833269

[10] Lamberton PH, Kabatereine NB, Oguttu DW, Fenwick A, Webster JP. Sensitivity and specificity of multiple Kato-Katz thick smears and a circulating cathodic antigen test for *Schistosoma mansoni* diagnosis pre- and post-repeated-praziquantel treatment. PLoS Negl Trop Dis. 2014;8: e3139. 25211217 10.1371/journal.pntd.0003139 PMC4161328

[11] Krauth SJ, Greter H, Stete K, Coulibaly JT, Traoré SI, Ngandolo BN, et al. All that is blood is not schistosomiasis: experiences with reagent strip testing for urogenital schistosomiasis with special consideration to very-low prevalence settings. Parasit Vectors. 2015;8:584. 26554822 10.1186/s13071-015-1165-y PMC4641389

[12] Weerakoon KG, McManus DP. Cell-free DNA as a diagnostic tool for human parasitic infections. Trends Parasitol. 2016;32:378–91. 26847654 10.1016/j.pt.2016.01.006

[13] Lu DB, Yu QF, Zhang JY, Sun MT, Gu MM, Webster JP, et al. Extended survival and reproductive potential of single-sex male and female *Schistosoma japonicum* within definitive hosts. Int J Parasitol. 2021;51:887–91. 33905765 10.1016/j.ijpara.2021.03.005

[14] Wang N, Peng HQ, Gao CZ, Cheng YH, Sun MT, Qu GL, et al. In vivo efficiency of praziquantel treatment of single-sex *Schistosoma japonicum* aged three months old in mice. Int J Parasitol Drugs Drug Resist. 2022;20:129–34. 36403362 10.1016/j.ijpddr.2022.11.002 PMC9771832

[15] Carson JP, Ramm GA, Robinson MW, McManus DP, Gobert GN. Schistosome-induced fibrotic disease: the role of hepatic stellate cells. Trends Parasitol. 2018;34:524–40. 29526403 10.1016/j.pt.2018.02.005

[16] Zhong H, Dong B, Zhu D, Fu Z, Liu J, Jin Y. Sja-let-7 suppresses the development of liver fibrosis via *Schistosoma japonicum* extracellular vesicles. PLOS Pathog. 2024;20: e1012153. 38598555 10.1371/journal.ppat.1012153 PMC11034668

[17] Reinholdt C, Winkelmann F, Koslowski N, Reisinger EC, Sombetzki M. Unisexual infection with *Schistosoma mansoni* in mice has the potential to boost the immune response against eggs after challenge infection. Front Immunol. 2023;14:1125912. 36923416 10.3389/fimmu.2023.1125912 PMC10009330

[18] Tang X, Qu GL, Dai J, Ji W, Zhou Y, Xu Y, et al. The study on mice hepatic fibrosis caused by adult unisexual *Schistosoma japonicum* infection. J Pathog Biol. 2022;17:796–801 (In Chinese).

[19] Sombetzki M, Koslowski N, Rabes A, Seneberg S, Winkelmann F, Fritzsche C, et al. Host defense versus immunosuppression: Unisexual infection with male or female *Schistosoma mansoni* differentially impacts the immune response against invading cercariae. Front Immuno. 2018;9:861. 10.3389/fimmu.2018.00861 PMC5930291 29743881

[20] Winkelmann F, Rabes A, Reinholdt C, Koslowski N, Koczan D, Reisinger EC, et al. Sex-specific modulation of the host transcriptome in the spleen of *Schistosoma mansoni*-infected mice. Front Cell Infect Microbiol. 2022;12: 893632. 35865813 10.3389/fcimb.2022.893632 PMC9294737

[21] Koslowski N, Sombetzki M, Loebermann M, Engelmann R, Grabow N, Osterreicher CH, et al. Single-sex infection with female *Schistosoma mansoni* cercariae mitigates hepatic fibrosis after secondary infection. PLoS Negl Trop Dis. 2017;11: e0005595. 28542175 10.1371/journal.pntd.0005595 PMC5453606

[22] He X, Wang Y, Fan X, Lei N, Tian Y, Zhang D, et al. A schistosome miRNA promotes host hepatic fibrosis by targeting transforming growth factor beta receptor III. J Hepatol. 2020;72:519–27. 31738999 10.1016/j.jhep.2019.10.029

[23] Wang L, Liao Y, Yang R, Yu Z, Zhang L, Zhu Z, et al. Sja-miR-71a in Schistosome egg-derived extracellular vesicles suppresses liver fibrosis caused by schistosomiasis via targeting semaphorin 4D. J Extracell Vesicles. 2020;9:1785738. 32944173 10.1080/20013078.2020.1785738 PMC7480424

[24] von Bülow V, Lichtenberger J, Grevelding CG, Falcone FH, Roeb E, Roderfeld M. Does *Schistosoma mansoni* facilitate carcinogenesis? Cells. 2021;10:1982. 34440754 10.3390/cells10081982 PMC8393187

[25] Jain S. Can *Schistosoma japonicum* infection cause liver cancer? J Helminthol. 2025;99: e11. 39924660 10.1017/S0022149X24000762

[26] Apari P, Földvári G. How do trematodes induce cancer? A possible evolutionary adaptation of an oncogenic agent transmitted by flukes. Evol Appl. 2025;18: e70070. 39845579 10.1111/eva.70070 PMC11751881

[27] Smout MJ, Laha T, Chaiyadet S, Brindley PJ, Loukas A. Mechanistic insights into liver-fluke-induced bile-duct cancer. Trend Parasitol. 2024;40:1183–96. 10.1016/j.pt.2024.10.012 PMC12212973 39521672

[28] Chuah C, Jones MK, Burke ML, McManus DP, Gobert GN. Cellular and chemokine-mediated regulation in schistosome-induced hepatic pathology. Trends Parasitol. 2014;30:141–50. 24433721 10.1016/j.pt.2013.12.009

[29] Corstjens P, de Dood CJ, Knopp S, Clements MN, Ortu G, Umulisa I, et al. Circulating anodic antigen (CAA): a highly sensitive diagnostic biomarker to detect active *Schistosoma* infections-improvement and use during SCORE. Am J Trop Med Hyg. 2020;103(1):50–7. 32400344 10.4269/ajtmh.19-0819 PMC7351307

[30] Sousa-Figueiredo JC, Betson M, Kabatereine NB, Stothard JR. The urine circulating cathodic antigen (CCA) dipstick: a valid substitute for microscopy for mapping and point-of-care diagnosis of intestinal schistosomiasis. PLoS Negl Trop Dis. 2013;7: e2008. 23359826 10.1371/journal.pntd.0002008 PMC3554525

[31] Langenberg MCC, Hoogerwerf MA, Koopman JPR, Janse JJ, Kos-van Oosterhoud J, Feijt C, et al. A controlled human *Schistosoma mansoni* infection model to advance novel drugs, vaccines and diagnostics. Nat Med. 2020;26:326–32. 32066978 10.1038/s41591-020-0759-x

